# Clinical analysis of aqueductal stenosis in patients with hydrocephalus in a Kenyan setting

**DOI:** 10.11604/pamj.2017.26.106.11050

**Published:** 2017-02-28

**Authors:** Loyal Poonamjeet Kaur, Nderitu Joseph Munyiri, Wekesa Vincent Dismus

**Affiliations:** 1 Department of Surgery, University of Nairobi, Kenya; 2 Department of Surgery, University of Nairobi, Kenya

**Keywords:** Aqueductal stenosis, hydrocephalus, Kenya

## Abstract

**Introduction:**

Aqueductal stenosis is the commonest cause of congenital hydrocephalus. The scope of this paper is to highlight the disease burden of hydrocephalus attributed to aqueductal stenosis which still remains unknown in our setting.

**Methods:**

In a descriptive cross-sectional study, 258 records of patients diagnosed with hydrocephalus were analyzed after ethical approval from Kenyatta National Hospital- University of Nairobi (KNH-UON) ethics and research committee from January 2010 to May 2016. Patients with a diagnosis of hydrocephalus due to aqueductal stenosis were included in this study. Patients age, sex, mode of delivery, associated comorbidities, presenting complaints, neurosurgical intervention performed, Kafarnosky score were recorded. Data were divided into 2 sets based on the patient's age i.e. whether < 1 year or > 12 years. Data were recorded on google data collection form and analyzed using Google spreadsheets.

**Results:**

Out of 258 cases of hydrocephalus, 52 had aqueductal stenosis. Male to female sex ratio for this condition was 3:2. There were 25 cases < 1year and 27 cases > 12 years old who were diagnosed with hydrocephalus due to aqueductal stenosis. Associated conditions were bilateral congenital talipes equinovarus, spina bifida, Arnold Chairi malformations, meningitis and HIV. The presenting complaints differed according to the age groups. Neurosurgical interventions included Endoscopic Third Ventriculostomy (ETV) in 21 cases, insertion of Ventriculoperitoneal (VP) shunt and ETV were done in 3 cases while the rest had only insertion of VP shunt. The Kafanosky score improve from < 50 pre-op to 19 cases achieving a score of 100, six months post-op.

**Conclusion:**

Aqueductal stenosis contributes a significant burden of morbidity in patients with hydrocephalus. Clinical presentation differs according to patients age. Accurate diagnosis and treatment remain a cardinal to improving patient outcome.

## Introduction

Aqueductal stenosis (AS) is a pathological condition causing triventricular obstructive hydrocephalus which requires a clinical and radiological diagnosis. Patients develop symptoms like headache, cognitive and gait impairment, and urinary incontinence [1]. Clinical presentation, neuro-radiological identification, and management of hydrocephalus secondary to aqueductal stenosis are specific [2]. Treatment options for hydrocephalus due to aqueductal stenosis includes insertion of ventriculopertitoneal shunt (VP) and Endoscopic Third ventriculostomy (ETV) [3–6]. It is the scope of this paper to focus on the disease burden of aqueductal stenosis for a setting for which it remains largely unknown.

## Methods

In a descriptive cross-sectional study, 258 records of patients at the Kenyatta National Hospital diagnosed with hydrocephalus were analyzed after ethical approval from Kenyatta National Hospital- University of Nairobi (KNH-UON) ethics and research committee from January 2010 to May 2016. Patients with a diagnosis of hydrocephalus due to aqueductal stenosis were included in this study. The variables collected include: patients age, sex, mode of delivery, associated comorbidities, presenting complaints, neurosurgical intervention performed, Kafarnosky score. Data were divided into 2 sets based on the patient's age i.e. whether < 1 year or > 12 years. Data were recorded on google data collection form and analyzed using Google spreadsheets.

## Results

In a sample study of 258 cases of hydrocephalus, aqueductal stenosis was identified as a cause of hydrocephalus in 52 cases (20%). The male to female sex ratio for this condition was 3:2. There were 25 cases (9.6%) of aqueductal stenosis in patients < 1year, of which 15 cases presented at birth with only 6 having undergone caesarean section; and 10 cases presented before the age of 6 months. 3 out of the 25 cases of aqueductal stenosis in <1 year old age group were premature. There were 27 (10.4%) cases of patients who were > 12 years old who were diagnosed with hydrocephalus due to aqueductal stenosis.

**Associated conditions:** These were almost exclusively seen in the age group of < 1 years. In 3 cases there was associated bilateral CTEV and spina bifida, 5 cases had associated spina bifida alone, 1 case had bilateral CTEV and 1 case of Arnold Chiari Type 2 malfomation with aqueductal stenosis. Meningitis was a co-morbidity in 6 cases while HIV was present in 3 cases. There was only 1 case of Arnold Chiari Type 1 malformation as an associated co-morbidity in the age group > 12 years.

**Figure 1:**
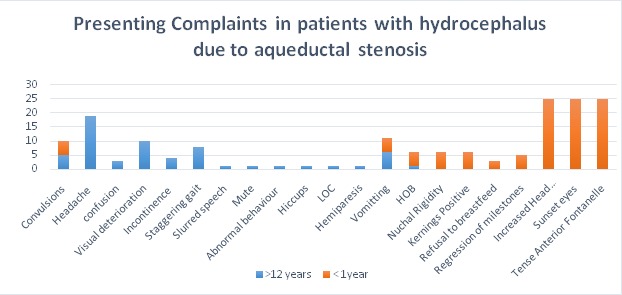
Common presenting complaints in patients with hydrocephalus due to aqueductal stenosis in a Kenyan setting. The most common complaints are: increase in head circumference, tense anterior fontanelle and sunset eyes in <1 year and headache, loss of vision, abnormal gait, convulsions, Vomiting stool and urine incontinence

**Presenting complaints:** For patients who were less than 1 years the main presenting complaints were increase in head circumference, tense anterior fontanelle and sunset eyes in all. Generalized tonic clonic seizures were seen in 5 cases, vomiting in 5 cases, regression of milestones in 5 cases, refusal to breastfeed in 3 cases; hotness of body, nuchal rigidity, and positive Kernings sign were seen in 6 cases. The median head circumference was 48.5cm for neonates who presented at birth and for 48.75cm those who presented before 6 months of age. For patients who were > 12 years the main presenting complaint was headache (19 cases), followed by deterioration of vision (10 cases), abnormal gait (8 cases), convulsions (5 cases), Vomitting ( 5 cases) stool and urine incontinence (4 cases). Other less common presenting complaints were confusion, loss of consciousness, slurred speech, hemiparesis, mutism, abnormal behavior and hiccups (Figure 1).

**Figure 2:**
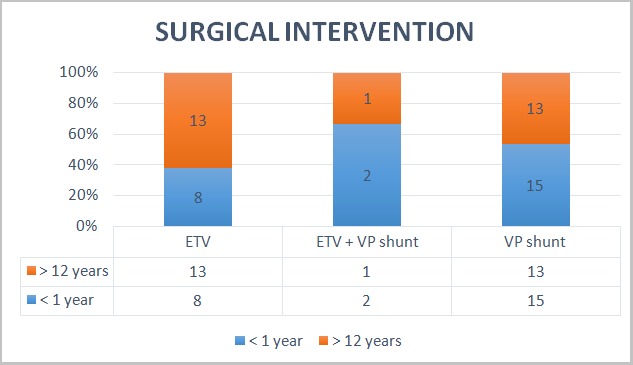
Neurosurgical interventions including Endoscopic third ventriculostomy (ETV) and Ventriculoperitoneal (VP) shunting carried out for patients with hydrocephalus due to aqueductal stenosis in the two age groups. In some patients VP shunt was placed before ETV was done

**Neurosurgical interventions:** As shown in Figure 2 below, majority of the patients (28 cases) had insertion of ventriculoperitoneal shunt (VPS). For those aged >12 years, 13 out of the 27 patients had Endoscopic Third Ventriculostomy (ETV) carried out and 1 had both ETV and VPS shunt insertion. For those who were < 1year old, 8 patients underwent ETV alone while 2 cases had both ETV and VP shunt insertion.

**Figure 3:**
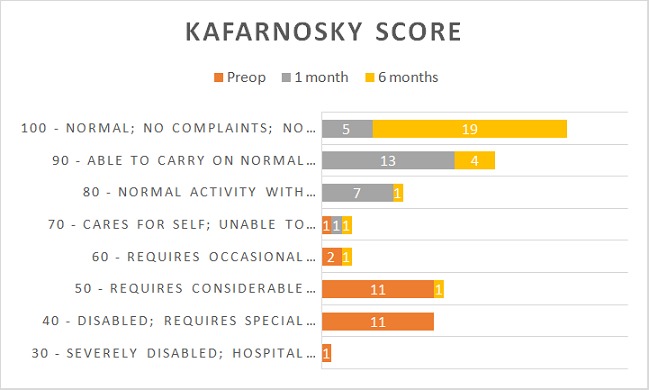
Kafarnosky score of patients aged > 12 years who were diagnosed with hydrocephalus due to aqueductal stenosis. This figure compares the Kafanosky score pre and post operatively

**Kafarnosky score:** The Kafarnosky score for patients who were 12 years and above was estimated preoperative, 1 month post-operative and 6 month post-operative (Figure 3). Majority of the patients preoperatively had a Kafarnosky score of 60 and below. The Kafarnosky score improved markedly in 6 month post-operatively with 19 out of the 27 patients having a score of 100.

## Discussion

Aqueductal stenosis is the commonest cause of congenital hydrocephalus. There has been paucity of data regarding the prevalence of this condition in our setting. There were 52 cases out of the 258 (20%) cases of hydrocephalus that had aqueductal stenosis as a cause of hydrocephalus, with a rate off 9.6% having congenital aqueductal stenosis and rate of 10% in adults. This is lower compared to reported cases of 30-40% with a varying prevalence of 15-60% of hydrocephalus in children and 10-40% of cases in adults [7, 8]. There was a bimodal distribution seen with age with one peak being before the age of 1 years and the second peak being after 12 years which compares with previously documented data [9]. The male to female ratio in this study of 3:2 differs from that documented of 14:13 [9, 10]. The presenting complaint for hydrocephalus differed according to the age of presentation. Early onset aqueductal stenosis was mainly associated with increase in head circumference, tense anterior fontanelle, sunset eyes. The main complaints for late onset were headache, visual deterioration, urine incontinence, abnormal gait, abnormal behavior. Both groups had convulsions and vomiting. These results are synchronous with previous studies [11]. Tremors have been reported in previous studies as a presenting complaint but this was not seen in this study [12]. Procedures available in the treatment of hydrocephalus include ventriculoperitoneal shunt and more recently Endoscopic Third ventirulostomy. The documented success rate for ETV is 75% [13] and is reported to have a shorter hospital stay compared to shunt insertion [14].

## Conclusion

Aqueductal stenosis contributes a significant burden of morbidity in patients with hydrocephalus. Clinical presentation differs according to patients´ age. Accurate diagnosis and treatment remain a cardinal to improving patient outcome.

### What is known about this topic

- Aqueductal stenosis has significant morbidity in patients with hydrocephalus;
- Prevalence and presenting complaints for aqueductal stenosis have been extensively studied in other settings;
- 2 modalities of intervention for aqueductal stenosis include ventriculoperitoneal shunting and endoscopic third ventriculostomy.

### What this study adds

- Prevalence of Aqueductal stenosis in the Kenyan setting;
- Comparison of Kafarnosky score pre-operatively and post-operatively in Kenyan setting;
- Prevalence and presenting complaints follow a bimodal distribution of age.
